# An upper bound for the pseudoisotopy stable range

**DOI:** 10.1007/s00208-016-1504-0

**Published:** 2016-12-01

**Authors:** Oscar Randal-Williams

**Affiliations:** Centre for Mathematical Sciences, Wilberforce Road, Cambridge, CB3 0WB UK

## Abstract

We prove that the pseudoisotopy stable range for manifolds of dimension 2*n* can be no better than $(2n-2)$. In order to do so, we define new characteristic classes for block bundles, extending our earlier work with Ebert, and prove their non-triviality. We also explain how similar methods show that $\mathrm {Top}(2n)/\mathrm {O}(2n)$ is rationally $(4n-5)$-connected.

For a smooth manifold *M*, possibly with boundary, the space of smooth pseudoisotopies (also known as concordances) is $P(M) := \mathrm {Diff}(M \times [0,1] \,\mathrm {rel}\, M \times \{0\})$, that is, the space of diffeomorphisms of the cylinder $M \times [0,1]$ which keep one end fixed. There is a canonical map

0.1 $$\begin{aligned} P(M) \longrightarrow P(M \times I) \end{aligned}$$

given by crossing with the interval *I* (and unbending corners), and the (smooth) *pseudoisotopy stable range* is the function

$$\begin{aligned} \phi (n) := \max \{k \in \mathbb {N}\,\vert \, \text {(0.1) is}\,\, k\text {-connected for all manifolds }\,M\,\text { of dimension}\,\ge n\}. \end{aligned}$$

The main theorem concerning this function is due to Igusa [16], and says that

$$\begin{aligned} \phi (n) \ge \min \left\{ \tfrac{n-7}{2}, \tfrac{n-4}{3}\right\} . \end{aligned}$$

In this note we establish the following upper bound for this function.

## Theorem A

$\phi (2n) \le 2n-2$ as long as $2n \ge 6$.

To explain our approach, let $W_{g,1} := \#^g S^n \times S^n {\setminus } \mathrm {int}(D^{2n})$ with $2n \ge 6$, and consider the fibration sequence

0.2 $$\begin{aligned} \frac{\widetilde{\mathrm {Diff}}_\partial (W_{g,1})}{{\mathrm {Diff}}_\partial (W_{g,1})} \longrightarrow B\mathrm {Diff}_\partial (W_{g,1}) \overset{i}{\longrightarrow }B\widetilde{\mathrm {Diff}}_\partial (W_{g,1}) \end{aligned}$$

from the classifying space of the group of diffeomorphisms of $W_{g,1}$ to the classifying space of the group of block diffeomorphisms of $W_{g,1}$. The rational cohomology of $B\mathrm {Diff}_\partial (W_{g,1})$ has been computed for $g \gg 0$ by Galatius and the author in [12, 13]; the rational cohomology of $B\widetilde{\mathrm {Diff}}_\partial (W_{g,1})$ has been computed for $g \gg 0$ by Berglund and Madsen in [1, 2] and in a forthcoming revision of [2]. Ebert and the author have shown in [8] that the map *i* is surjective on rational cohomology in the stable range.

Our approach to Theorem A is motivated by forthcoming work of Berglund and Madsen, in which they show that the map induced by *i* on rational cohomology is injective in degrees $* < 2n$ and $g \gg 0$, and more importantly for our current purpose they show that this is sharp, in the following sense.

## Proposition B

(Berglund–Madsen) For $g \gg 0$,

0.3 $$\begin{aligned} \mathrm {Ker}(i^* : H^{2n}(B\widetilde{\mathrm {Diff}}_\partial (W_{g,1});\mathbb {Q}) \rightarrow H^{2n}(B{\mathrm {Diff}}_\partial (W_{g,1});\mathbb {Q})) \ne 0. \end{aligned}$$

This has implications for the Serre spectral sequence of (0.2), and it is this that we shall exploit to prove Theorem A. As Proposition B is central to our argument, and its proof is not yet available, in Sects. 2 and 3 we will give an independent proof of it, which works for all $g \ge 1$ and does not require the computation of both groups. It consists of defining Mumford–Morita–Miller classes for block bundles, which extend those that we have already defined with Ebert in [8], and then showing that a certain such class—namely $\tilde{\kappa }_{e^2} - \tilde{\kappa }_{p_n}$, which is easily seen to lie in the kernel (0.3)—is not trivial. The construction of these classes and their non-triviality may be of interest independently of Theorem A.

Finally, in Sect. 4 we show how similar methods can be used to show that the space $\mathrm {Top}(2n)/\mathrm {O}(2n)$ is rationally $(4n-5)$-connected as long as $2n > 4$.

## Proof of Theorem A

By the work of Weiss–Williams [22, Theorem A], there is a certain map

1.1 $$\begin{aligned} \frac{\widetilde{\mathrm {Diff}}_\partial (W_{g,1})}{{\mathrm {Diff}}_\partial (W_{g,1})} \longrightarrow \Omega ^\infty (S^\infty _+ \wedge _{\mathbb {Z}/2} \Omega \mathbf {Wh}^{\mathrm {Diff}}_s(W_{g,1})) \end{aligned}$$

which is $(\phi (2n)+1)$-connected. The ($\mathbb {Z}/2$-)spectrum $\mathbf {Wh}^{\mathrm {Diff}}_s(W_{g,1})$ is the 1-connected cover of the (smooth) Whitehead spectrum $\mathbf {Wh}^{\mathrm {Diff}}(W_{g,1})$, which in turn is related to Waldhausen’s algebraic *K*-theory of spaces by a (split) cofibre sequence of spectra

1.2 $$\begin{aligned} \Sigma ^\infty _+ W_{g,1} \longrightarrow \mathbf {A}(W_{g,1}) \longrightarrow \mathbf {Wh}^{\mathrm {Diff}}(W_{g,1}). \end{aligned}$$

This identification requires the stable parameterised *h*-cobordism theorem [20].

Our strategy is then as follows. We use a theorem of Hsiang–Staffeldt to compute the spectrum cohomology $H^*(\mathbf {Wh}^{\mathrm {Diff}}(W_{g,1});\mathbb {Q})$ in degrees $* \le 2n$. We take care to compute this *as a representation of the mapping class group* $\Gamma _{g,1}$ of $W_{g,1}$, in terms of the standard representation

$$\begin{aligned} H_g := H_n(W_{g,1};\mathbb {Q}) \end{aligned}$$

of $\Gamma _{g,1}$. The spectrum cohomology of $S^\infty _+ \wedge _{\mathbb {Z}/2} \Omega \mathbf {Wh}^{\mathrm {Diff}}_s(W_{g,1})$ is then given by truncating, desuspending, and taking $\mathbb {Z}/2$-invariants, and the cohomology of $\Omega ^\infty (S^\infty _+ \wedge _{\mathbb {Z}/2} \Omega \mathbf {Wh}^{\mathrm {Diff}}_s(W_{g,1}))$ is the free graded-commutative algebra on the result.

We now suppose for a contradiction that $\phi (2n) \ge 2n-1$, so the map (1.1) is 2*n*-connected and hence we have a computation of the rational cohomology of $\frac{\widetilde{\mathrm {Diff}}_\partial (W_{g,1})}{{\mathrm {Diff}}_\partial (W_{g,1})}$ in degrees $* \le 2n-1$, as a $\Gamma _{g,1}$-module. We then study the Serre spectral sequence for (0.2), and derive a contradiction.

### Rational homology of the Whitehead spectrum

We shall use Corollary 1.2 of Hsiang–Staffeldt [15], which shows that

$$\begin{aligned} H_*(\mathbf {A}(W_{g,1});\mathbb {Q}) = \pi _*(\mathbf {A}(W_{g,1})) \otimes \mathbb {Q}\cong (K_*(\mathbb {Z}) \otimes \mathbb {Q}) \oplus (\Sigma \bar{K}_{ab}) \end{aligned}$$

where *K* is a minimal model for the dga $C_*(\Omega W_{g,1};\mathbb {Q})$, $\bar{K}$ denotes the augmentation ideal, which inherits the structure of a graded Lie algebra with bracket given by $[x,y] := x \cdot y - (-1)^{\vert x \vert \cdot \vert y \vert } y \cdot x$, and $\bar{K}_{ab} = \bar{K}/[\bar{K}, \bar{K}]$ is the abelianisation of this graded Lie algebra.

As $W_{g,1}$ is a suspension, the homology of $\Omega W_{g,1}$ is the tensor algebra on the vector space $H_g[n-1]$. In particular it is a free (non-commutative) algebra, so is quasi-isomorphic to $C_*(\Omega W_{g,1};\mathbb {Q})$, and we may take $K=H_*(\Omega W_{g,1};\mathbb {Q})$ with trivial differential. It follows that $\bar{K}_{ab}$ is the augmentation ideal of the free graded commutative algebra on $H_g[n-1]$, that is

$$\begin{aligned} \bar{K}_{ab} = (H_g[n-1])&\oplus \left( \begin{array}{ll} \mathrm {Sym}^2(H_g)[2n-2] &{}\quad \text {if} \,\,n\,\, \text {is odd}\\ \wedge ^2(H_g)[2n-2] &{}\quad \text {if} \,\,n\,\, \text {is even} \end{array} \right) \\&\oplus \,(\text {terms of degree} \ge 3n-3). \end{aligned}$$

Let us write

$$\begin{aligned} U := {\left\{ \begin{array}{ll} \mathrm {Sym}^2(H_g) &{} \quad \text {if}\,\, n\,\, \text {is odd}\\ \wedge ^2(H_g) &{} \quad \text {if} \,\,n\,\, \text {is even.} \end{array}\right. } \end{aligned}$$

Then we have

$$\begin{aligned} H_*(\mathbf {A}(W_{g,1});\mathbb {Q}) \cong (K_*(\mathbb {Z}) \otimes \mathbb {Q}) \oplus (H_g[n]) \oplus (U[2n-1]) \end{aligned}$$

in degrees $* \le 2n$. Applying the cofibre sequence (1.2), we obtain

$$\begin{aligned} H_*(\mathbf {Wh}^{\mathrm {Diff}}(W_{g,1});\mathbb {Q}) \cong (\tilde{K}_*(\mathbb {Z}) \otimes \mathbb {Q}) \oplus (U[2n-1]) \end{aligned}$$

in degrees $* \le 2n$. The rational homology of $\mathbf {Wh}^{\mathrm {Diff}}_s(W_{g,1})$ is therefore the same, as it is already 1-connected. Thus, dualising, we have

$$\begin{aligned} H^*(S^\infty _+ \wedge _{\mathbb {Z}/2} \Omega \mathbf {Wh}^{\mathrm {Diff}}_s(W_{g,1});\mathbb {Q}) \cong ((\tilde{K}_{*-1}(\mathbb {Z}) \otimes \mathbb {Q}) \oplus (U[2n-2]))^{\mathbb {Z}/2} \end{aligned}$$

in degrees $* \le 2n-1$, for some involution. It follows from Farrell–Hsiang [10] (which considers the case $g=0$) that this involution acts as $-1$ on $\tilde{K}_{*-1}(\mathbb {Z}) \otimes \mathbb {Q}$, so this summand does not contribute to the invariants. Thus

$$\begin{aligned} H^*(S^\infty _+ \wedge _{\mathbb {Z}/2} \Omega \mathbf {Wh}^{\mathrm {Diff}}_s(W_{g,1});\mathbb {Q}) \cong (U[2n-2])^{\mathbb {Z}/2} \end{aligned}$$

in degrees $* \le 2n-1$, for some involution on *U*. Taking the free graded-commutative algebra on this, it follows that

$$\begin{aligned} H^*(\Omega ^\infty (S^\infty _+ \wedge _{\mathbb {Z}/2} \Omega \mathbf {Wh}^{\mathrm {Diff}}_s(W_{g,1}));\mathbb {Q}) \cong \mathbb {Q}[0] \oplus (U[2n-2])^{\mathbb {Z}/2} \end{aligned}$$

in degrees $$* \le 2n-1$$.

### The Serre spectral sequence argument

The Serre spectral sequence for the fibration (0.2) takes the form

$$\begin{aligned} E_1^{p,q} = H^p\left( B\widetilde{\mathrm {Diff}}_\partial (W_{g,1}); H^q\left( \tfrac{\widetilde{\mathrm {Diff}}_\partial (W_{g,1})}{{\mathrm {Diff}}_\partial (W_{g,1})};\mathbb {Q}\right) \right) \Longrightarrow H^{p+q}(B{\mathrm {Diff}}_\partial (W_{g,1});\mathbb {Q}). \end{aligned}$$

Under the assumption that $\phi (2n) \ge 2n-1$ we have identified the coefficients in degrees $q \le 2n-1$, to be $\mathbb {Q}$ for $q=0$ and to be $V:=U^{\mathbb {Z}/2}$ for $q=2n-2$. In order for (0.3) to be possible, we must therefore have a non-trivial differential

$$\begin{aligned} d^{2n-1} : H^1(B\widetilde{\mathrm {Diff}}_\partial (W_{g,1});V) \longrightarrow H^{2n}(B\widetilde{\mathrm {Diff}}_\partial (W_{g,1});\mathbb {Q}). \end{aligned}$$

In particular, the source must be non-trivial. Note that $H^1(B\widetilde{\mathrm {Diff}}_\partial (W_{g,1});V)$ is a summand of $H^1(B\widetilde{\mathrm {Diff}}_\partial (W_{g,1});U)$, so the following will give a contradiction.

#### Proposition 1.1

$$H^1(B\widetilde{\mathrm {Diff}}_\partial (W_{g,1});U)=0$$ for $$g \gg 0$$.

#### Proof

The action of $\Gamma _{g,1}$ on $H_n(W_{g,1};\mathbb {Z})$ preserves the intersection form, determining a homomorphism

$$\begin{aligned} \Gamma _{g,1} \longrightarrow {\left\{ \begin{array}{ll} O_{g,g}(\mathbb {Z}) &{}\quad \text {if }\, n \,\text { is even}\\ Sp_{2g}(\mathbb {Z}) &{}\quad \text {if }\, n \,\text { is odd}. \end{array}\right. } \end{aligned}$$

This is onto if *n* is even or $n=1,3,7$, but for the remaining odd *n* its image is the finite-index subgroup—often denoted $\Gamma _g(1,2) \le Sp_{2g}(\mathbb {Z})$ in the theory of theta functions—of those symplectic matrices which preserve the standard quadratic form, cf. [2, Example 4.2]. Let us write *G* for the algebraic group $O_{g,g}$ or $Sp_{2g}$, depending on the parity of *n*, and $A_g \le G(\mathbb {Z})$ for the image of this homomorphism. As $Sp_{2g}$ and $SO_{g,g}$ are connected semisimple algebraic groups defined over $\mathbb {Q}$, it follows from a theorem of Borel–Harish-Chandra [5, Theorem 7.8] that $A_g$ is a lattice in $G(\mathbb {R})$, and hence by the Borel Density Theorem [3] that $A_g$ is Zariski dense in $G(\mathbb {R})$, so also in $G(\mathbb {C})$.

Consider the fibration sequence

$$\begin{aligned} B\widetilde{\mathfrak {Tor}}_{g,1} \longrightarrow B\widetilde{\mathrm {Diff}}_\partial (W_{g,1}) \longrightarrow BA_g, \end{aligned}$$

where $B\widetilde{\mathfrak {Tor}}_{g,1}$ is defined to be the homotopy fibre. By [2, Proposition 4.1] we have

$$\begin{aligned} H^1(B\widetilde{\mathfrak {Tor}}_{g,1};\mathbb {Q}) \cong {\left\{ \begin{array}{ll} H_g &{}\quad n \equiv 3 \mod 4\\ 0 &{}\quad \text {else} \end{array}\right. } \end{aligned}$$

so if $n \not \equiv 3 \mod 4$ then $H^1(A_g; U) \rightarrow H^1(B\widetilde{\mathrm {Diff}}_\partial (W_{g,1});U)$ is an isomorphism, and if $n \equiv 3 \mod 4$ then we have an exact sequence

$$\begin{aligned} 0 \longrightarrow H^1(A_g; U) \longrightarrow H^1(B\widetilde{\mathrm {Diff}}_\partial (W_{g,1});U) \longrightarrow (H_g \otimes U)^{A_g}. \end{aligned}$$

In the case $n \equiv 3 \mod 4$, *n* is odd and Zariski density of $A_g \le Sp_{2g}(\mathbb {C})$ implies that the complexification of $(H_g \otimes \mathrm {Sym}^2(H_g))^{A_g}$ is $(H_g \otimes \mathrm {Sym}^2(H_g) \otimes \mathbb {C})^{Sp_{2g}(\mathbb {C})}$, which is contained in $(H_g^{\otimes 3} \otimes \mathbb {C})^{Sp_{2g}(\mathbb {C})}$ and so vanishes by standard invariant theory (for which we refer to [11, §F.2]).

It remains to show that $H^1(A_g; U)=0$. The representation *U* is arithmetic, so a theorem of Borel [4, Theorem 1] can be used to identify this with $H^1(A_g; \mathbb {Q}) \otimes U^{A_g}$ as long as $g \gg 0$; see [9, Proposition 3.9] for a statement of this result adapted to our situation. Hence it is enough to show the vanishing of $U^{A_g}$.

If *n* is odd then $U^{A_g}$ is $\mathrm {Sym}^2(H_g)^{A_g}$, whose complexification is the same as $\mathrm {Sym}^2(H_g \otimes \mathbb {C})^{Sp_{2g}(\mathbb {C})}$ by Zariski density, and this vanishes by standard invariant theory. If *n* is even then $U^{A_g}$ is $\wedge ^2(H_g)^{A_g}$, whose complexification is $\wedge ^2(H_g \otimes \mathbb {C})^{O_{g,g}(\mathbb {C})}$, which also vanishes by standard invariant theory (noting that $O_{g,g}(\mathbb {C}) \cong O_{2g}(\mathbb {C})$).

$$\square $$

## Characteristic classes of block bundles

We should like to give a proof of Proposition B, as it does not require the entire corpus [1, 2, 12, 13] and beyond to see that the kernel (0.3) is non-trivial. We shall show that this kernel is non-trivial by producing an explicit element in it, which will be described in terms of generalised Mumford–Morita–Miller classes. If $(\pi : E \rightarrow \vert K \vert , \mathcal {A})$ is a smooth oriented block bundle with fibre a closed *d*-manifold *M* (we refer to [8, Section 2] for this notation), in [8, Section 3] Ebert and the author have associated to it

(i) a Leray–Serre spectral sequence $H^p(\vert K \vert , \mathcal {H}^q(M)) \Rightarrow H^{p+q}(E)$, and hence a fibre-integration map $\pi _!(-) : H^{k+d}(E) \rightarrow H^k(\vert K \vert )$,
(ii) a transfer map $\mathrm {trf}^*_\pi : H^*(E) \rightarrow H^*(\vert K \vert )$ of Becker–Gottlieb type,
(iii) a *stable* vertical tangent bundle $T_\pi ^s E \rightarrow E$,

such that if $(\pi : E \rightarrow \vert K \vert , \mathcal {A})$ arises from a smooth fibre bundle then these data reduce to those coming from the bundle structure. In the case $d=2n$, we then employed the following ruse: If $\pi$ came from a smooth fibre bundle with 2*n*-dimensional fibres, so there was an *unstable* vertical tangent bundle $T_\pi E$, then we would have $e(T_\pi E)^2 = p_n(T_\pi E)$, and $\pi _!(e(T_\pi E) \cdot -) = \mathrm {trf}^*_\pi (-) : H^*(E) \rightarrow H^*(\vert K \vert )$. Therefore, for a monomial $p_I$ in Pontrjagin classes, if we define

$$\begin{aligned} \tilde{\kappa }_{p_I}(\pi ) := \pi _!(p_I(T_\pi ^s E)) \quad \quad \quad \tilde{\kappa }_{e p_I} := \mathrm {trf}^*_\pi (p_I(T_\pi ^s E)) \end{aligned}$$

then these classes restrict to the usual $\kappa _{p_I}$ and $\kappa _{ep_I}$ on fibre bundles, and these give all generalised Mumford–Morita–Miller classes on fibre bundles.

By way of apology for this ruse, we add to the list above

(iv) an Euler class $e(T_\pi E) \in H^{d}(E;\mathbb {Z})$.

(Of course $e(T_\pi E)$ is merely notation: there is no *d*-dimensional bundle $T_\pi E$ of which it is the Euler class.) Using this Euler class, we may then define

$$\begin{aligned} \tilde{\kappa }_{e^i p_I}(\pi ) := \pi _!(e(T_\pi E)^i \cdot p_I(T_\pi ^s E)) \in H^*(\vert K \vert ; \mathbb {Z}). \end{aligned}$$

The symbol $\tilde{\kappa }_{ep_I}$ has the same meaning as before, by Lemma 2.2 (iv) below.

The existence of this Euler class is a consequence of the Fibre Inclusion Theorem of [7] (or rather its proof, which constructs a canonical such class), and the fact that the homotopy fibre of $\pi$ is homotopy equivalent to a Poincaré duality space of dimension *d*, namely *M* [8, Proposition 2.8]. As the construction is quite pretty, let us describe it.

### Construction 2.1

Embed $\vert K \vert$ into $\mathbb {R}^k$ for some $k \gg 0$, and let $B'$ be a closed regular neighbourhood, so that there is a retraction $r : B' \rightarrow \vert K \vert$. Let $B=D(B')$ be the double of $B'$, a closed smooth manifold. This has a retraction $s : D(B') \rightarrow B'$, and let $p : X \rightarrow B$ be the Hurewicz fibration obtained by turning $\pi$ into a fibration $\pi ^f : E^f \rightarrow \vert K \vert$ and pulling it back along *rs*. As *B* and the fibre of *p* are Poincaré duality spaces, of dimensions *k* and *d* respectively, *X* is too [14], of dimension $(d+k)$. But $X \times _B X = p^*(X) \rightarrow X$ is also a fibration over a Poincaré duality space with Poincaré duality fibre, so is again a Poincaré duality space, of dimension $(2d+k)$. Writing $\Delta : X \rightarrow X \times _B X$ for the fibrewise diagonal map, which admits an umkehr map $\Delta _!$ as source and target are both Poincaré, we define

$$\begin{aligned} e(T_p X) := \Delta ^* \Delta _!(1) \in H^{d}(X;\mathbb {Z}). \end{aligned}$$

We then define $e(T_\pi E)$ by restriction along $E \subset E^f \subset X\vert _{B'} \subset X$.

It is easy to see that the class so obtained is independent of all choices, and it is shown in [7, §4] that it restricts to the Euler class on the fibre *M*. The definition given in [7, §4] seems to differ by a sign, but it does not, by Lemma 2.2 (i) below.

### Lemma 2.2

The Euler class defined enjoys the following properties:

(i) If *d* is odd then $2e(T_\pi E)=0 \in H^*(E;\mathbb {Z})$,
(ii) if $(\pi : E \rightarrow \vert K \vert , \mathcal {A})$ arises from a smooth fibre bundle with vertical tangent bundle $T_\pi E$, then $e(T_\pi E)$ agrees with the Euler class of the vertical tangent bundle,
(iii) if there is a map $r : E \rightarrow M$ such that $\pi \times r : E \rightarrow \vert K \vert \times M$ is a homotopy equivalence, then $e(T_\pi E) = r^*(e(TM))$,
(iv) the equation $\pi _!(e(T_\pi E) \cdot -) = \mathrm {trf}_\pi ^*(-) : H^*(E;\mathbb {Z}) \rightarrow H^*(\vert K \vert ;\mathbb {Z})$ is satisfied.

### Proof

For (i), consider the involution $\tau$ of $X \times _B X$ which interchanges the two factors. When *d* is odd, this has degree $-1$, and so $\tau ^* \Delta _! = -\Delta _!$. On the other hand $\Delta ^* \tau ^* = \Delta ^*$, so $e(T_\pi E)=-e(T_\pi E)$.

For (ii), note that if $(\pi : E \rightarrow \vert K \vert , \mathcal {A})$ arises from a smooth fibre bundle then in Construction 2.1 we do not need to replace it by a fibration. The resulting $p : X \rightarrow B$ is a smooth fibre bundle with vertical tangent bundle $T_p X$, and the map $\Delta : X \rightarrow X \times _B X$ is a smooth embedding with normal bundle $T_p X$. Hence $\Delta ^*\Delta _!(1)$ is the Euler class of $T_p X$, which restricts to the Euler class of $T_\pi E$.

For (iii), if such an *r* exists then the fibration $p : X \rightarrow B$ admits a similar fibre homotopy trivialisation, $p \times \rho : X \overset{\sim }{\rightarrow }B \times M$. Then $X \times _B X \simeq B \times M \times M$ and the map $\Delta$ is given by the identity map on *B* and the diagonal map on *M*. Hence $\Delta ^*\Delta _!(1) = 1 \otimes e(TM)$.

For (iv), we must involve ourselves in the details of the construction of the transfer in [7], with which we assume the reader is familiar. We begin by constructing a commutative diagramIn this diagram, *B* is a Poincaré duality space and *p* is a Hurewicz fibration with fibre $F \simeq M^{d}$ (obtained as in Construction 2.1). *W* is a smooth oriented manifold of dimension $(d+\ell )$ with boundary, which is homotopy equivalent to *M*, and $p'$ is a smooth fibre bundle (obtained from the Closed Fibre Smoothing Theorem of [7]). The map $p''$ is obtained as the fibrewise double of $p'$, and is a smooth oriented fibre bundle with closed fibres. Finally, the horizontal arrows express each left-hand space as a (fibrewise) retract of the right-hand space.

For a fibration $p : S \rightarrow T$ with fibre homotopy equivalent to a finite CW complex, and a fibrewise map $f : S \rightarrow S$, let us write $\mathrm {trf}_{p, f}^* : H^*(S) \rightarrow H^*(T)$ for the associated transfer map. This is the map denoted $\tau ^f$ in [7]. When $f = \mathrm {Id}_S$, we shorten this to $\mathrm {trf}_p^*$.

By the definition of the transfer in [7, §6], we have $\mathrm {trf}_{p}^* = \mathrm {trf}_{p'', vuts}^* s^* t^*$. By the construction of the transfer for smooth fibre bundles in [7, §5], if we write

$$\begin{aligned} \delta = (\mathrm {Id}_{X''}, vuts) : X'' \longrightarrow X'' \times _B X''\\ d = (\mathrm {Id}_{X''}, \mathrm {Id}_{X''}) : X'' \longrightarrow X'' \times _B X'' \end{aligned}$$

then we have $\mathrm {trf}_{p'', vuts}^*(-) = p''_!(\delta ^*(d_!(1)) \cdot -)$. Thus the map $\mathrm {trf}_{p}^*(-)$ is $p''_!(\delta ^*(d_!(1))\cdot s^* t^*(-))=(p t s)_!(\delta ^*(d_!(1))\cdot s^* t^*(-))$, which we may write as $p_!((t s)_!(\delta ^*(d_!(1)) \cdot -)$, so we will be done if $(t s)_!(\delta ^*(d_!(1)))$ is equal to the class $e(T_{p}X)$ defined by Construction 2.1. Consider the homotopy cartesian squaresof Poincaré duality spaces, to which Lemma 2.3 below applies and shows that

$$\begin{aligned} (ts)_! \delta ^* = \Delta ^* (\mathrm {Id} \times vu)^* (ts \times \mathrm {Id})_! \quad \quad (ts \times \mathrm {Id})_! d_!(ts)^* = (\mathrm {Id} \times ts)^* \Delta _!. \end{aligned}$$

(The signs can be determined by restricting each square to a single fibre over *B*.) Thus, writing $1=(ts)^*(1)$, we have

$$\begin{aligned} (ts)_!\delta ^*d_!(ts)^*(1)&= \Delta ^* (\mathrm {Id} \times vu)^* (ts \times \mathrm {Id})_!d_!(ts)^*(1)\\&= \Delta ^* (\mathrm {Id} \times vu)^* (\mathrm {Id} \times ts)^* \Delta _!(1) = \Delta ^*\Delta _!(1) \end{aligned}$$

which is $e(T_{p}X)$, as required. $\square$

### Lemma 2.3

Consider a homotopy cartesian squareof oriented Poincaré duality spaces. Then $g_! u^* = \pm v^* f_!$.

The sign ambiguity is unavoidable under the given hypotheses: changing the orientation of *B*, say, does not change $g_! u^*$, but changes $v^* f_!$ by a sign.

### Proof

Let us write *a* for the formal dimension of *A*, and so on. We assume some familiarity with the notion of Poincaré embeddings, for which we refer to [17] for details. It is enough to prove the identity for the larger squareBy this device, we may suppose [17, Lemma 3.1] that *f* admits the structure of a Poincaré embedding, with complement *K* and normal spherical fibration $\xi$ of dimension $(d-b-1)$. Let $u^*\xi \rightarrow A$ denote the pulled back spherical fibration, and $v^* K \rightarrow C$ denote the homotopy pullback of the map $K \rightarrow D$ along *v*. There is then a homotopy commutative cubein which the bottom face is homotopy cocartesian, and the vertical faces are all homotopy cartesian. It follows by Mather’s Second Cube Theorem [18, Theorem 25] that the top face is also homotopy cocartesian. We therefore have a map

$$\begin{aligned} C \simeq A \cup _{u^* \xi } v^*K \longrightarrow A/u^*\xi = \mathrm {Th}(u^*\xi ) \end{aligned}$$

by collapsing $v^*K$, and similarly for *K*. This gives a homotopy commutative diagramwhich in cohomology yields the required equation. From this point of view, the sign ambiguity arises from the two possible choices of Thom class for $u^*\xi$: the one compatible with the fundamental classes [*C*] and [*A*], or the pullback of the one compatible with [*D*] and [*B*]. $\square$

## Proof of Proposition B

We can extend the definition of the classes $\tilde{\kappa }_{e^i p_I}$ to block bundles having fibres $W_{g,1}$ by filling in a disc in each fibre, giving a new block bundle with fibre $W_g := W_{g,1} \cup _\partial D^{2n} = \#^g S^n \times S^n$. There are therefore defined universal characteristic classes $\tilde{\kappa }_{e^i p_I} \in H^*(B\widetilde{\mathrm {Diff}}_\partial (W_{g,1});\mathbb {Q})$, by the proof of [8, Theorem 3.4].

In particular, we have a class $\tilde{\kappa }_{e^2} - \tilde{\kappa }_{p_n} \in H^{2n}(B\widetilde{\mathrm {Diff}}_\partial (W_{g,1});\mathbb {Q})$ which vanishes in $H^{2n}(B\mathrm {Diff}_\partial (W_{g,1});\mathbb {Q})$, because $e^2 = p_n$ on the total space of a smooth fibre bundle. Proposition B is an immediate consequence of the following.

### Proposition 3.1

For each $g \ge 1$ and each $n \ge 3$ there is a block bundle $(\pi : E \rightarrow \vert K \vert , \mathcal {A})$ with fibre $W_{g,1}$, such that

(i) $$\tilde{\kappa }_{e^2}(\pi )=0 \in H^{2n}(\vert K \vert ;\mathbb {Q})$$,
(ii) $$\tilde{\kappa }_{p_n}(\pi ) \ne 0 \in H^{2n}(\vert K \vert ;\mathbb {Q})$$.

Therefore $$\tilde{\kappa }_{e^2} - \tilde{\kappa }_{p_n} \ne 0 \in H^{2n}(B\widetilde{\mathrm {Diff}}_\partial (W_{g,1});\mathbb {Q})$$.

### Proof

From Lemma 2.2 (iii) it follows that the $\tilde{\kappa }_{e^i}$ vanish for all $i>0$ on all fibre homotopically trivial block bundles. We will therefore construct $\pi$ to be fibre homotopically trivial, guaranteeing that $\tilde{\kappa }_{e^2}(\pi )=0$.

We will use the (space-level) surgery fibration of Quinn [19], which following the discussion in [1, Section 3.2], in particular equation (43), may be put in the form

$$\begin{aligned} \left( \frac{\mathrm {hAut}_\partial (W_{g,1})}{\widetilde{\mathrm {Diff}}_\partial (W_{g,1})}\right) _{(1)} \longrightarrow \mathrm {map}_*(W_{g,1}/\partial W_{g,1}, G/O)_{(1)} \overset{\sigma }{\longrightarrow }\mathbb {L}_{2n}(\mathbb {Z})_{(1)}. \end{aligned}$$

Thus to construct a fibre homotopically trivial block bundle over *B* (with some triangulation) it is enough to give a map $f : B \rightarrow \mathrm {map}_*(W_{g,1}/\partial W_{g,1}, G/O)_{(1)}$ and a nullhomotopy of $\sigma \circ f$.

For simplicity of exposition we restrict to the case $n=2k$. We let $B=S^n \times S^n$, write $a, b \in H^n(B;\mathbb {Q})$ for a hyperbolic basis, and write $e_1, f_1, \ldots , e_g, f_g \in H^n(W_{g,1}, \partial W_{g,1};\mathbb {Q})$ for a hyperbolic basis. Write the *n*th Hirzebruch *L*-polynomial as $\mathcal {L}_n = A p_n + B p_{n/2}^2$ modulo other Pontrjagin classes, for some constants *A* and *B*. It is well-known that $A \ne 0$, and less well-known but true [21, Lemma A.1] that $B \ne 0$.

As the composition

$$\begin{aligned} p: G/O \overset{i}{\longrightarrow }BO \overset{\prod p_i}{\longrightarrow }\prod _{i=1}^\infty K(\mathbb {Z}, 4i) \end{aligned}$$

has homotopy fibre with finite homotopy groups, we claim that may find a map *f* whose adjoint $\hat{f} : (B \times W_{g,1}, B \times \partial W_{g,1}) \rightarrow (G/O, *)$ composed with *i* gives a class

$$\begin{aligned} \xi \in KO^0(B \times W_{g,1}, B \times \partial W_{g,1}) \end{aligned}$$

which has $p_{n/2}(\xi ) = C \cdot (a \otimes e_1 + b \otimes f_1)$, $p_n(\xi ) = -\tfrac{2B C^2}{A}\cdot a\cdot b \otimes e_1 \cdot f_1$, and all other rational Pontrjagin classes zero, for some constant $C \ne 0$. To establish this claim, let the map

$$\begin{aligned} \varphi : (B \times W_{g,1}, B \times \partial W_{g,1}) \longrightarrow \left( \prod _{i=1}^\infty K(\mathbb {Z}, 4i), *\right) \end{aligned}$$

classify the pair of relative cohomology classes $L \cdot (a \otimes e_1 + b \otimes f_1)$ and $-\tfrac{2B L^2}{A}\cdot a\cdot b \otimes e_1 \cdot f_1$, for some integer $L \ne 0$ large enough that these classes are integral. For each $N > 0$ consider the map $\phi _N : \prod _{i} K(\mathbb {Z}, 4i) \rightarrow \prod _{i} K(\mathbb {Z}, 4i)$ which multiplies by $N^i$ on $K(\mathbb {Z}, 4i)$. The diagramthen admits a dotted lift $\hat{f}$ for *N* large enough, as the universal obstructions to finding such a lift lie in the cohomology of $\prod _{i} K(\mathbb {Z}, 4i)$ with finite coefficients, and are therefore annihilated (on each skeleton) by some $\phi _N$. The resulting map $\hat{f}$ gives $p_{n/2}(\xi ) = L \cdot N^{n/2} \cdot (a \otimes e_1 + b \otimes f_1)$, $p_n(\xi ) = -\tfrac{2B L^2}{A} \cdot N^n \cdot a\cdot b \otimes e_1 \cdot f_1$, and all other Pontrjagin classes zero, as required (with $C=L \cdot N^{n/2}$).

We must show that the composition

$$\begin{aligned} B=S^n \times S^n \overset{f}{\longrightarrow }\mathrm {map}_*(W_{g,1}/\partial W_{g,1}, G/O)_{(1)} \overset{\sigma }{\longrightarrow }\mathbb {L}_{2n}(\mathbb {Z})_{(1)} \end{aligned}$$

is nullhomotopic, but we shall allow ourselves to precompose *f* with self-maps $k_N : S^n \times S^n \rightarrow S^n \times S^n$ having degree $N \ne 0$ on both factors (such a precomposition preserves the form of Pontrjagin classes which has been arranged above). With this in mind, it is enough to show that

$$\begin{aligned} \sigma \circ f = 0 \in [B, \mathbb {L}_{2n}(\mathbb {Z})] \otimes \mathbb {Q}. \end{aligned}$$

This group may be identified with $H^{4*}(B;\mathbb {Q})$. If $n \equiv 0 \mod 4$ then the component of degree $n=2k=4\ell$ is identified with the Künneth factor of

$$\begin{aligned} \tfrac{1}{8}\mathcal {L}_{3\ell }(\xi ) \in H^{12\ell }(B \times W_{g,1}, B \times \partial W_{g,1};\mathbb {Q}) \end{aligned}$$

in $H^{4\ell }(B;\mathbb {Q}) \cong H^{4\ell }(B;\mathbb {Q}) \otimes H^{8\ell }(W_{g,1}, \partial W_{g,1};\mathbb {Q})$. But $\mathcal {L}_{3\ell }(\xi )=0$ by observation, as only $p_{4\ell }(\xi )$ and $p_{2\ell }(\xi )$ are non-zero. Whatever the class of *n* modulo 4, the component of degree $2n=4k$ is identified with the Künneth factor of

$$\begin{aligned} \tfrac{1}{8}\mathcal {L}_{2k}(\xi ) \in H^{8k}(B \times W_{g,1}, B \times \partial W_{g,1};\mathbb {Q}) \end{aligned}$$

in $H^{4k}(B;\mathbb {Q})$. But by construction

$$\begin{aligned} \mathcal {L}_{2k}(\xi )= A \cdot \left( -\tfrac{2B C^2}{A}\cdot a\cdot b \otimes e_1 \cdot f_1\right) + B \cdot (C \cdot (a \otimes e_1 + b \otimes f_1))^2=0. \end{aligned}$$

We therefore obtain a map *f*, with $\sigma \circ f$ nullhomotopic and $i \circ \hat{f}$ classifying a vector bundle $\xi '$ having $p_{n/2}(\xi ') = D \cdot (a \otimes e_1 + b \otimes f_1)$, $p_n(\xi ') = -\tfrac{2B D^2}{A}\cdot a\cdot b \otimes e_1 \cdot f_1$, and all other Pontrjagin classes zero, for some constant $D \ne 0$. (The constant will have changed when we precomposed the original choice of *f* with the maps $k_N$.) The associated block bundle $\pi : E \rightarrow \vert K \vert \approx B$ has $T_v^s E \simeq _s TE - \pi ^*TB = \epsilon ^{2n}+\xi '$ (see [8, Lemma 3.3]) and so

$$\begin{aligned} \tilde{\kappa }_{p_n}(\pi ) = \pi _!(p_n(T_v^s E)) = \pi _!(p_n(\xi ')) = -\tfrac{2B D^2}{A}\cdot a\cdot b \ne 0 \end{aligned}$$

as required.

It is not difficult to adapt the above argument to work for $n = 2k+1$. The essential point is that if we write $\mathcal {L}_n = A p_n + B p_{\tfrac{n-1}{2}}p_{\tfrac{n+1}{2}}$ modulo all other Pontrjagin classes, then $A \ne 0$ and again by [21, Lemma A.1] $B \ne 0$. We then take $B=S^{2k-1} \times S^{2k+3}$ and proceed as above. $\square$

## Rational connectivity of $\mathrm {Top}(2n)/\mathrm {O}(2n)$

Our goal in this section is to show how similar techniques to those we have been using imply the following.

### Theorem 4.1

$$\pi _*(B\mathrm {Diff}_\partial (D^{2n})) \otimes \mathbb {Q}=0$$ for $$1 \le * \le 2n-5$$.

This extends the analogous calculation of Farrell–Hsiang [10], which established the same result in degrees $1 \le * \le \phi (2n)$. By smoothing theory we have a homotopy equivalence $B\mathrm {Diff}_\partial (D^{2n}) \simeq \Omega ^{2n}_0 (\mathrm {Top}(2n)/\mathrm {O}(2n))$ as long as $2n > 4$, from which we deduce that

### Corollary 4.2

$\mathrm {Top}(2n)/\mathrm {O}(2n)$ is rationally $(4n-5)$-connected as long as $n>2$.

On the other hand, it has been shown by Weiss [21] that

$$\begin{aligned} H^{4n}(B\mathrm {Top}(2n);\mathbb {Q}) \longrightarrow H^{4n}(B\mathrm {O}(2n);\mathbb {Q}) \end{aligned}$$

has nontrivial kernel for $n \gg 0$ (namely, the class $e^2 - p_n$), so $\mathrm {Top}(2n)/\mathrm {O}(2n)$ is *not* rationally $(4n-1)$-connected.

### Proof of Theorem 4.1

Let $W_g = \#^g S^n \times S^n {\setminus } \mathrm {int}(D^{2n})$, with $2n \ge 6$, and choose a collar $[0,1) \times \partial W_{g,1} \subset W_{g,1}$ and a disc $D^{2n} \subset (0,1) \times \partial W_{g,1}$. The map

4.1 $$\begin{aligned} \frac{\widetilde{\mathrm {Diff}}_\partial (D^{2n})}{{\mathrm {Diff}}_\partial (D^{2n})} \longrightarrow \frac{\widetilde{\mathrm {Diff}}_\partial (W_{g,1})}{{\mathrm {Diff}}_\partial (W_{g,1})} \end{aligned}$$

is $(2n-4)$-connected, by Morlet’s lemma of disjunction [6, Corollary 3.2]. Consider the fibration

$$\begin{aligned} \frac{\widetilde{\mathrm {Diff}}_\partial (W_{g,1})}{{\mathrm {Diff}}_\partial (W_{g,1})} \longrightarrow B{\mathrm {Diff}}_\partial (W_{g,1}) \overset{i}{\longrightarrow }\widetilde{\mathrm {Diff}}_\partial (W_{g,1}). \end{aligned}$$

Up to isotopy any diffeomorphism $\varphi$ representing an element of $\pi _1(B\widetilde{\mathrm {Diff}}_\partial (W_{g,1}))$ may be supposed to be equal to the identity on the collar: the map (4.1) is then preserved by that induced by $\varphi$, and it then follows that $\varphi$ acts trivially on $H^*\left( \tfrac{\widetilde{\mathrm {Diff}}_\partial (W_{g,1})}{{\mathrm {Diff}}_\partial (W_{g,1})};\mathbb {Q}\right)$ in the range of degrees $* \le 2n-4$ where the map (4.1) is a cohomology injection. Hence the Serre spectral sequence

$$\begin{aligned} H^p\left( B\widetilde{\mathrm {Diff}}_\partial (W_{g,1}); H^q\left( \tfrac{\widetilde{\mathrm {Diff}}_\partial (W_{g,1})}{{\mathrm {Diff}}_\partial (W_{g,1})};\mathbb {Q}\right) \right) \Longrightarrow H^{p+q}(B\mathrm {Diff}_\partial (W_{g,1});\mathbb {Q}) \end{aligned}$$

associated to this fibration has a product structure in this range of degrees. But Berglund–Madsen have shown that the map

$$\begin{aligned} i^* : H^*(B\widetilde{\mathrm {Diff}}_\partial (W_{g,1});\mathbb {Q}) \longrightarrow H^*(B{\mathrm {Diff}}_\partial (W_{g,1});\mathbb {Q}) \end{aligned}$$

is an isomorphism in degrees $* \le 2n-1$ as long as $g \gg 0$. This result will appear soon in a revision of [2]. It follows that $H^q\left( \tfrac{\widetilde{\mathrm {Diff}}_\partial (W_{g,1})}{{\mathrm {Diff}}_\partial (W_{g,1})};\mathbb {Q}\right) =0$ for $1 \le q \le 2n-4$, and hence by the map (4.1) that $H^q\left( \tfrac{\widetilde{\mathrm {Diff}}_\partial (D^{2n})}{{\mathrm {Diff}}_\partial (D^{2n})};\mathbb {Q}\right) =0$ for $1 \le q \le 2n-5$.

On the other hand, the surgery fibration sequence shows that $\frac{\mathrm {hAut}_\partial (D^{2n})}{\widetilde{\mathrm {Diff}}_\partial (D^{2n})}$ is rationally acyclic, and $\mathrm {hAut}_\partial (D^{2n}) \simeq *$ by the Alexander trick, so $B\widetilde{\mathrm {Diff}}_\partial (D^{2n})$ is rationally acyclic. Boundary connect-sum makes this into an *H*-space, so it has trivial rational homotopy groups. Thus $\widetilde{\mathrm {Diff}}_\partial (D^{2n})$ has finitely-many components, and each one is rationally acyclic. The group ${\mathrm {Diff}}_\partial (D^{2n})$ has the same components, and so the quotient $\tfrac{\widetilde{\mathrm {Diff}}_\partial (D^{2n})}{{\mathrm {Diff}}_\partial (D^{2n})}$ is rationally homotopy equivalent to $B{\mathrm {Diff}}_\partial (D^{2n})_0$, the classifying space of the component of the identity in $B{\mathrm {Diff}}_\partial (D^{2n})$. It follows from the above that its rational cohomology, and hence rational homotopy, vanishes in degrees $1 \le * \le 2n-5$. $\square$
